# Improved local control using higher dose SBRT in metastatic sarcoma patients

**DOI:** 10.1186/s13014-025-02719-3

**Published:** 2025-09-08

**Authors:** Mattias Hedman, Elia Rossi, Emmy Dalqvist, Kristin Karlsson, Christina Linder-Stragliotto

**Affiliations:** 1 Department of Radiation Oncology, Karolinska University Hospital, Stockholm, Sweden; 2 Department of Breast Sarcoma and Endocrine Tumors, Karolinska University Hospital, Stockholm, Sweden; 3 Department of Nuclear Medicine and Medical Physics, Karolinska University Hospital, Stockholm, Sweden; 4 Department of Oncology-Pathology, Karolinska Institutet, Stockholm, Sweden; 5 Department of Molecular Medicine and Surgery, Karolinska Institutet, Stockholm, Sweden

**Keywords:** Stereotactic Body Radiotherapy (SBRT), Soft tissue sarcoma (STS) Metastasis, High-dose fraction

## Abstract

**Background:**

Stereotactic Body Radiotherapy (SBRT) has been proven to be a safe and effective alternative to surgery in patients with metastatic primary sarcoma. However, data describing tumor response in relation to the given radiotherapy dose is lacking. Therefore, this study aims at analyzing efficacy and dose–response relationship in a retrospective cohort.

**Methods:**

Patients with metastatic sarcoma treated with ablative SBRT and followed up at the Karolinska University Hospital between 2008 and 2021 were included. SBRT was delivered using an inhomogeneous dose distribution resulting in higher median doses within the planning target volume (PTV) than the dose prescribed. Local control (LC), progression-free survival (PFS), overall survival (OS), adverse events and dose–response relationship were assessed. Statistical analysis was performed to identify variables that correlate to outcome.

**Results:**

Forty-three patients with a total of 83 lesions were treated. The most frequent histology was leiomyosarcoma (44%). The most common site of metastases was the lung (84%), followed by the liver (11%). The median prescription dose was 45 Gy (range 30–56 Gy) delivered in 3 fractions (range 2–8) with a planned median CTV mean dose of 309 Gy in EQD_2_ with α/β = 3 Gy. The local control at 1-year, 2-year and 5-year from SBRT treatment was 97, 93 and 84%, respectively. For tumors with a planned mean CTV dose above EQD_2_ 278.8 Gy (corresponding to 60.3 Gy in 3 fractions) the 1, 2 and 5-year local control was 100, 100 and 93%, respectively. Tumors planned with a lower dose than EQD_2_ 278.8 Gy (α/β = 3 Gy) had a 1, 2 and 5-year local control of 90, 70 and 52%, respectively. The difference in local control between the high dose and low dose groups was statistically significant (*p* < 0.001). The median OS for all patients was 43 months. When respecting dose constraints, there were only limited number of mild side effects.

**Conclusion:**

In this analysis a strongly significant dose–response relationship with excellent LC rates and limited side effects for patients with metastatic lesions of sarcoma were seen. These results could be related to the inhomogeneous dose distribution of SBRT treatments utilized in this study.

## Background

Sarcoma is a rare mesenchymal tumor with various subtypes. In general, soft tissue sarcoma responds poorly to chemotherapeutic regimens making radiotherapy an important option when treating symptoms from metastatic disease [1]. Advanced-stage bone and soft tissue sarcoma represent a significant challenge due to a high incidence of relapses. In the case of recurrent disease in a limited number of locations, surgery is an important treatment option with improved overall survival [2]. Stereotactic body radiation therapy (SBRT) shows a high local control rate for various tumor types when used in the single or oligometastatic setting [3]. As a consequence, SBRT has been introduced as an important treatment option, especially for inoperable patients or if surgery is technically difficult. A growing number of studies have been published evaluating the efficacy of SBRT in treating metastatic sarcoma demonstrating that it achieves high local control rates and that it is well-tolerated and safe [4–17]. However, data on the dose–response relationship, especially in relation to the actual median dose delivered to individual tumors, are lacking. SBRT is often delivered using an inhomogeneous dose distribution resulting in higher median doses within the planning target volume (PTV) compared to the prescribed dose [18, 19]. Therefore, to accurately assess tumor response in relation to the radiation dose, additional information beyond the prescribed dose to the PTV is needed. There are only a few papers reporting dose–response relationship [5, 6, 12, 14], and description of quality assurance of given treatment is often lacking, making conclusions of the effect of radiotherapy dose on tumor and overall response difficult.

A retrospective study conducted over a decade ago at our institution, demonstrated high local control with minimal side effects for patients with metastatic sarcoma treated with SBRT between 1994 and 2005 [17]. The patient cohort was quite heterogenic regarding delivered doses and treatment fractions. Since then, several advancements in radiotherapy techniques have been implemented. Particularly, the introduction of online image-guided radiation therapy (IGRT) allows for better precision and higher dose prescriptions with the use of cone-beam computed tomography (CBCT) images at each treatment session. To evaluate the impact of these advancements we conducted another retrospective analysis on a more recent cohort of patients with the specific aim to study the relationship between radiotherapy dose and tumor response.

## Methods

### Patients

This retrospective cohort study includes all patients with metastatic soft-tissue or bone sarcoma treated with ablative SBRT at the Karolinska University Hospital between 2008 and 2021. Ablative treatment was defined as exceeding a planned and delivered radiotherapy dose of 60 Gy, as mean dose to clinical target volume (CTV), expressed in equivalent dose in 2 Gy fractions (EQD_2_) with α/β = 10 Gy (α/β10). The year 2008 coincides with the introduction of the oncology information system (OIS) ARIA (Varian medical systems, Palo Alto, USA) at our institution, which was used to identify all patients. Patients lacking clinical follow-up data were excluded from the analysis.

### Treatment technique

Patients were fixated with a custom-fitted vacuum cushion either in a stereotactic body frame (Elekta, Stockholm, Sweden) or in combination with a wingboard (Civco, Iowa, USA). An abdominal compression plate was used for targets located caudal to the carina. Computed tomography (CT) simulation imaging for lung and upper abdomen targets was performed with both 3D and 4D CT scans. Magnetic resonance imaging (MRI) in treatment position was used for targets in the spine. Treatment-plans were created on a CT in a random breathing phase and delivered with static fields, conformal arc or VMAT. Treatment was delivered using TrueBeam, Clinac 2100 and during 2008 Varian CI600C (Varian Medical Systems inc., Palo Alto, CA, USA). Daily image-guidance with CBCT was used for patients treated after mid-2009, while out-of-room verification CT was used for earlier treated patients (5 treatments).

### Target definition

Clinical target volume (CTV) was defined with no margin to the gross tumor volume (GTV). Respiratory motion was addressed by either incorporation of the tumor breathing motion in the PTV margin calculation or the creation of an ITV if the tumor movement was considered irregular, unusually large or overlapping with the diaphragm in the breathing cycle. PTV was generated by adding an individual margin to the CTV, resulting in approximately 10 mm in the longitudinal direction and 5 mm in the transversal direction, or using an isotropic margin of 5 mm to the ITV.

### Dose prescription and evaluation

According to our routine, prescribed doses generally range between 45–56 Gy delivered in 3, 5 or 8 fractions, with a risk-adapted regimen depending on proximity to organs at risk (OARs). Our preferred dose is 45 Gy in three fractions in order to deliver the highest biological dose to the target. The dose is prescribed to the PTV periphery to approximately the 67% isodose line in relation to the maximum dose, aiming for optimal coverage of the PTV while minimizing exposure to nearby OARs and respecting dose constraints. The tumor dose assessed was the planned mean dose to the CTV for each target lesion, recalculated into EQD_2_, using both α/β10 and α/β = 3 Gy (α/β3), to be able to compare treatments with different fractionations. Recalculating with α/β3 was done to maintain consistency with our previously published material [17] and is also in line with recent data showing that sarcoma has an α/β below 5 Gy [20]. The α/β10 was included to compare our doses with doses in several publications that present doses in biologically effective dose (BED) calculated with α/β10 [5, 7–9, 13, 16].

The dose–response relationship between the mean CTV dose and local tumor control was evaluated comparing tumors with a planned CTV mean dose equal or above our preferred prescription of 45 Gy in three fractions to those receiving a lower dose, usually as a compromise to spare organs at risk.

### Follow-up and definition of response

Local control was assessed on repeated CT scans, MR images, or CT-PET images after treatment and based on the radiology report. The radiological evaluation after SBRT categorized the response as follows: complete response (CR) indicated the absence of any visible tumor, partial response (PR) denoted a visible reduction in size compared to the lesion before treatment, and stable disease (SD) indicated no significant change in size, all of which were categorized as local control (LC). Progressive disease (PD) was defined as a visible increase in the cross-sectional tumor diameter or recurrence within the PTV, both of which result in local failure (LF).

The time to LF was measured from the date of the final SBRT treatment to the occurrence of PD. Progression-Free Survival (PFS) was defined as the time span from the SBRT treatment to the documentation of progression at any location or death. Overall Survival (OS) was defined as the time interval between the SBRT treatment and either the date of death from any cause or the last follow-up date.

Treatment related toxicity was graded using Common Terminology Criteria for Adverse Events (CTCEA) version 5.0.

### Statistical methods

The Kaplan–Meier method was used to estimate time-to-event outcomes, including LF, PFS, and OS. Kaplan–Meier curves were generated for each of these endpoints, and the log-rank test was used to compare survival distributions across different subgroups.

Univariable Cox proportional hazards (PH) regression modeling was conducted to investigate the association between patient- and tumor-specific variables and PFS or LC (time to local failure). These models estimate hazard ratios (HR) with corresponding 95% confidence intervals (CI), providing a measure of the relative risk of progression associated with each parameter.

All statistical analyses were conducted using R, with the significance level set at 0.05.

The study was approved by the Swedish Ethical Review Authority (nr 2022–04561-01).

## Results

Forty-three patients, 23 women and 20 men, were included in the analysis. Patient characteristics are presented in Table 1. The median age at the time of the first treatment was 60 years, 64 for women and 55 for men. A total of 83 lesions were treated, ranging from 1 to 7 lesions per patient. Among these, one tumor was a case of re-irradiation. The median follow-up time per patient was 29.5 months, with a range of 3–132 months. Four tumors were excluded due to missing follow-up data. The most frequent histologies were leiomyosarcoma (19 patients), osteosarcoma (3 patients), synovial sarcoma (3 patients) and pleomorphic sarcoma (3 patients). The most common site of metastases was the lung (67 targets), followed by the liver (9 targets). Median clinical target volume (CTV) was 2.4 cm^3^ (range 0.13- 171.2 cm^3^). The median prescription dose was 45 Gy (range 30—56 Gy) and the median number of delivered fractions was 3 (range 2–8) with a planned median CTV mean dose of 309 Gy in EQD_2_ α/β3 (range 124–410 Gy), see Table 2. SBRT was administered for multiple lesions in 19 patients (44%), and in more than one treatment session in 11 patients (25%). Thirty-one patients (72%) had additional metastases alongside the ones targeted for SBRT at the time of their initial SBRT treatment. Additionally, 29 patients (66%) received chemotherapy either before, after, or both before and after undergoing SBRT.

**Table 1:** Patient characteristics

Characteristics	
**43 patients**	
*Gender*	
Male	20 (46%)
Female	23 (54%)
*Age*^*a*^* (years)*	
Mean	57.1
Median	60
Range	19–85
*Histology*	
Soft tissue sarcoma^b^	17 (40%)
Uterine sarcoma	10 (23%)
Osteosarcoma	3 (7%)
Ewing	2 (5%)
Nerve-derivated sarcoma^c^	4 (9%)
Other^d^	7 (16%)
*Primary tumor grade*	
High grade (G3-4)	28 (65%)
Low grade (G2)	7 (16%)
Other^e^	8 (19%)
*Previously or later treated with:*	
Surgery	25 (57%)
Chemotherapy	29 (66%)
Radiotherapy	32 (73%)
**57 treatments**	
*ECOG PS*	
0	36 (63%)
1	21 (37%)
*Number of known metastases at time of treatment*	
0	12 (21%)
1	15 (26%)
2–6	15 (26%)
> 6	15 (26%)
*Number of targets per SBRT treatment*	
1	37 (65%)
2	15 (26%)
3	4 (7%)
4	1 (2%)
**83 lesions**	
*CTV (cm*^*3*^*)*	
Mean	12.5
Median	2.4
Range	0.1–171.2
*Localization*	
Lung	70 (84%)
Liver	9 (11%)
Iliac artery	1 (1%)
Scapula	1 (1%)
Mediastinum	1 (1%)
Spine	1 (1%)

^a^If a patient was treated several times, the age at the first treatment is presented here

^b^Leiomyosarcoma (n = 10), synovial sarcoma (n = 3), liposarcoma (n = 2), solitary fibrous tumor (n = 1), alveolar soft part sarcoma (n = 1)

^c^Peripheral nerve sheath sarcoma (n = 2), chordoma (n = 1), neurofibrosarcoma (n = 1)

^d^Pleomorphic sarcoma (n = 3), GIST (n = 2), spindle cell sarcoma (n = 1), phyllodes tumor (n = 1)

^e^No information on tumor grade

**Table 2:** Prescribed and planned dose, both with EQD_2_ α/β3 and α/β10

Total dose (Gy)	No of fractions	α/β 10		α/β 3		No of tumors (%)
		Prescribed dose EQD_2_ (Gy)	Mean dose to CTV EQD_2_ (Gy) range	Prescribed dose EQD_2_ (Gy)	Mean dose to CTV EQD_2_ (Gy) range	
30	3	50.0	80.5	78.0	136.0	1 (1.2)
30	2	62.5	112.3	108.0	209.6	1 (1.2)
40	4	66.7	91.9–107.2	104.0	151.4–181.1	3 (3.6)
42	6*	59.5	100.0–107.1	84.0	156.0–169.1	2 (2.4)
36 + 7	4 + 1	66.9	80.3	100.4	124.4	1 (1.)
45	3	93.8	151.4–190.5	162.0	278.8–360.4	52 (62.7)
45	5	71.2	123.7	108.0	206.4	1 (1.2)
48	4	88.0	121.8–157.0	144.0	209.6–280.1	2 (2.4)
48	8	64.0	96.0–96.4	86.4	140.8–141.5	2 (2.4)
48	3	104.0	185.4	182.4	349.7	1 (1.2)
50	5	83.3	81.1–144.7	130.0	126.0–247.2	10 (12.0)
51	3	114.8	202.6–213.9	204.0	385.9–409.8	3 (3.6)
51.2	8	70.0	122.2	96.3	187.6	1 (1.2)
56	8	79.3	122.7–137.2	112.0	188.5–215.1	3 (3.6)

^*^Both tumors belonged to the same patient, and only 6 out of 8 fractions were delivered

**Fig. 1:**
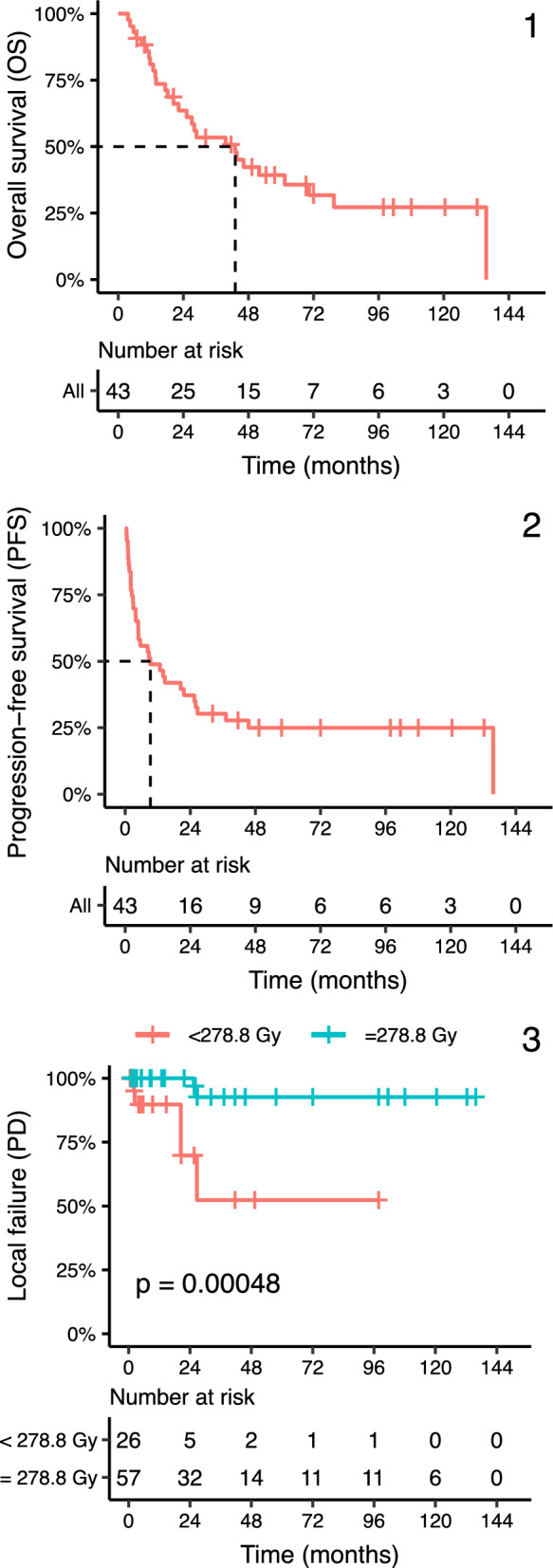
Kaplan–Meier curves for clinical outcomes. **1** Overall survival (OS) and (**2**) Progression-free survival (PFS) with median values indicated by dashed lines. **3** Local failure (LF) stratified by CTV mean dose (EQD_2_ < 278.8 Gy vs. ≥ 278.8 Gy (α/β3)). Numbers at risk are shown below each graph

The local control at 1-year and 2-years after SBRT treatment was 97 (CI: 93–100%) and 93% (CI: 86–100%), respectively. At 5-years, local control was 84% (CI: 73–96%). For tumors with a planned CTV mean dose equal or above our preferred prescription of 45 Gy in three fractions, which in our material equals an EQD_2_ 278.8 Gy (α/β3), the 1-year, 2-year and 5-year local control was 100 (CI: 100–100%), 100 (CI: 100–100%) and 93% (CI: 83–100%), respectively. Tumors planned with a lower CTV mean dose than EQD_2_ 278.8 Gy (α/β3) had a 1-year, 2-year and 5-year local control of 90 (CI: 77–100%), 70 (CI: 48–100%) and 52% (CI: 26–100%), respectively. The difference in local control between the high dose and low dose groups was statistically significant (*p* < 0.001). Kaplan–Meier curves for OS, PFS and LF are illustrated in Fig. 1.

The median PFS for all patients was 9.3 months (CI: 4.8–27). The 1-year, 2-year and 5-year PFS was 49 (CI: 36–66%), 37 (CI: 25–55%) and 25% (CI: 15–42%), respectively. On Cox PH regression univariable analysis, male gender (HR = 2.04 (CI: 1.24–3.35); *p* = 0.005), chemotherapy after SBRT (HR = 2.85 (CI: 1.12–7.27); *p* = 0.028), chemotherapy before and after SBRT (HR = 4.02 (CI: 2.22–7.29); *p* < 0.001), and two or more known metastasis at the time of SBRT (HR = 1.98 (CI: 1.01–3.88); *p* = 0.047) were all associated with worse PFS. Increased CTV mean dose was associated with better LC and the normalized HR for LC (time to local failure) was 0.397 and 0.408 when evaluating CTV mean dose in EQD2 (Gy) with α/β3 and α/β10, respectively, both with a significant *p* value (< 0.01). We did not see a correlation between neither localization of metastasis and PFS, nor tumor volume and LC. The median OS for all patients was 43 months (CI: 25–80 months), and the 1, 2 and 5-year survival was 81 (CI: 70–94%), 64 (CI: 50–80%) and 39 (CI: 29–51%), respectively.

Mild side effects were seen in 4 patients; with three cases of pneumonitis grade 1–2 and one case of pneumonitis grade 1 together with asymptomatic rib fractures. Three additional cases of rib fractures were seen on follow-up CT scans, however they reported no symptoms according to CTCEA. There was only one patient with higher-grade toxicity, a grade 5 bleeding reported among the early treated patients where an association with the SBRT treatment could not be excluded. This patient was re-irradiated two times and the approximate post-treatment reconstructed accumulated dose to the trachea had a point max of EQD_2_ 290 Gy and a volume of 9.8 cm^3^ received more than EQD_2_ 100 Gy (α/β3).

## Discussion

In this single-cohort retrospective analysis we present data that supports a dose–response relationship between planned CTV mean dose in EQD_2_ and local control when treating metastatic sarcoma with SBRT. The results show significantly better local control when the mean dose to the CTV is above 278.8 Gy in EQD_2_ α/β3. Overall, only mild grade 1–2 side effects in a limited number of patients were observed, concluding that the treatment was well tolerated. However, there was one grade five side effect, a lethal bleeding from an ulceration close to the remaining right bronchus a week after the last SBRT treatment. This patient had been treated with SBRT in two previous treatment sessions and had significant anatomical changes in the thorax making accurate dose-summations of the different treatment sessions difficult and imprecise, and tumor cells were found adjacent to the ulceration after autopsy. The treatment was performed before the result of the Nordic HILUS-trial [21] was presented and is not consistent with our current guidelines where strict dose constraints to central mediastinal structures are applied. Our interpretation is that an escalated dose is safe as long as dose constraints to the organs at risk are respected.

**Fig. 2:**
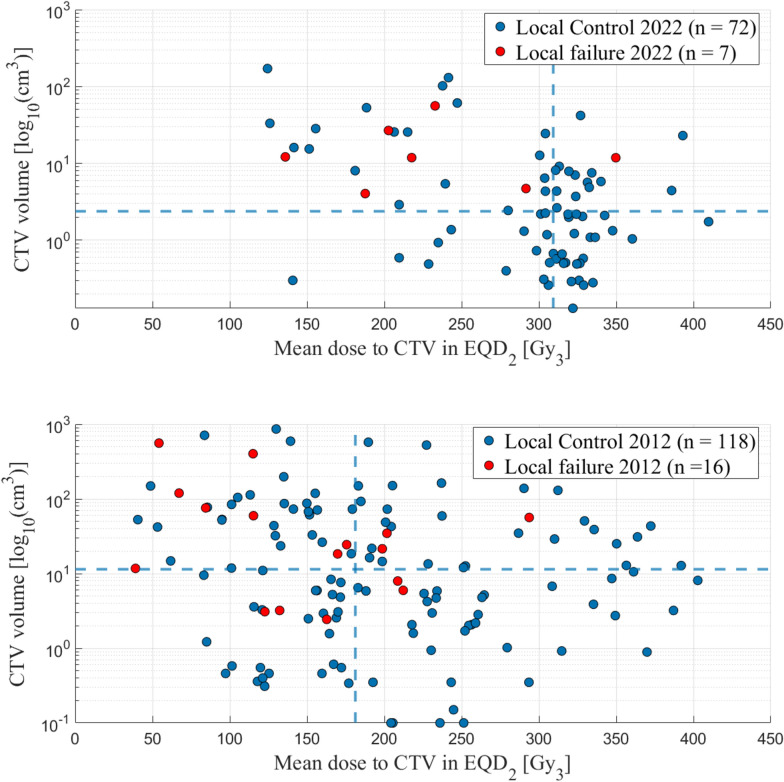
Comparison of the CTV volume in relation to the CTV mean dose in EQD_2_ (α/β3) in our current patient cohort (above) and in the previously published study (below). The lines represent the median CTV mean dose and the median CTV volume

One purpose of this study was to perform an update on the results for SBRT treatments of sarcoma in comparison with our previous data presented in the study from 2012 [17]. We conclude that we today deliver considerably higher tumor doses, median CTV mean dose 309 Gy versus 181 Gy in EQD_2_ (αβ3), in a more standardized manner regarding prescribed dose and fractionation. Over the years, we have established a routine for risk-adapted treatments, encompassing not only risk-adapted dose prescriptions and treatment planning, but also a structured approach to image guidance to ensure safe treatments. Our image-guidance protocol includes the use of isodose structures at the OAR tolerance dose levels, created from the treatment plan, during online CBCT matching for both tumor and OAR. This helps to ensure that the tolerance dose for the OAR is not exceeded. Additionally, a verification CBCT is performed after each treatment fraction to confirm tumor and OAR position, with the option to adapt the treatment plan for subsequent fractions if necessary in case of intra-fraction movement. Additionally, we treat smaller volumes, median 2.4 cm^3^ versus 11.4 cm^3^, indicating a stricter selection of patients (see Fig. 2). This is also reflected by the fact that only patients in good performance status (PS) of 0–1, according to the Eastern Cooperative Oncology Group (ECOG) Performance Status Scale, are included in the updated material. In the previous publication, PS was not available, and thus not evaluated. Consequently, survival is significantly better in the current cohort with a median OS of 43 months versus 26.3 months.

At our institution, SBRT is always delivered with an inhomogeneous dose to the target resulting in significantly higher mean doses to the CTV than the prescribed (see Table 2). Planned doses are seldom reported in other published materials making evaluations difficult and most studies only report prescribed dose to the target, adding to the uncertainty of the delivered dose. Consequently, the impact of radiotherapy dose on local control has remained unclear. As part of the project “Hypofractionated Treatment Effects in the Clinic (HyTEC)”, efforts were made to investigate the dose–response relationship following SBRT. Similar to our findings, the authors highlight the uncertainties in the dosimetric data, noting that most reviewed articles only provided information on the prescribed radiotherapy doses. However, two systematic reviews reported tumor control probability (TCP) models demonstrating improved local control rates with higher radiotherapy doses for metastases in the liver and spine [22, 23].

Our data indicate that increasing the dose to the target is important to improve the probability of local control. A limited number of previous studies evaluating the effect of SBRT on metastatic sarcoma have demonstrated a significant dose–response relationship when evaluating local control rates [5, 6, 12, 14]. In our study, increasing the CTV mean dose above EQD_2_ 278.8 Gy (α/β3), is beneficial for achieving excellent local control rates. The results support dose escalation, however, the planned radiotherapy doses in our study cohort are substantially higher than those prescribed in the other studies. We also demonstrate superior local control rates at 1 and 2 years.

Progressive disease was only seen in tumors with a CTV volume above the median, however there was no significant correlation between the CTV volume and LC in this patient-cohort. This aligns with most studies which report no significant correlation between tumor size and tumor control [4, 6, 8, 11]. One study on the other hand presents a marginally significant improved local progression-free survival related to tumor volume below 9 cm^3^ [12]. Regarding tumor size, one study correlates tumor diameter greater than 3 cm with poorer LC [14]. Another study reports worse LC and OS for tumors exceeding 4 cm in size [7], while a third study finds a correlation between worse LC and tumors larger than 5 cm [13]. Conversely, other studies present tumor size without a correlation with outcome [9, 10, 15]. There are thus conflicting results regarding the importance of tumor size, although the majority of data suggests that tumor size should not be a limitation for treatment. Tumor size might on the other hand affect the doses to organs at risk and subsequently the possibility to deliver ablative doses to the tumor.

## Limitations

Given the retrospective nature of this study it has several limitations. Radiological evaluations were not conducted at fixed time points for all patients and RECIST criteria were not applied in this retrospective study. Side effects were not collected systematically and reported according to CTCEA criteria. Mild side effects could go undetected or be missed, as they are difficult to correlate with the radiotherapy treatment. However, we are confident that our current follow-up protocol detects all severe and moderate side effects.

Additionally, due to the limited sample size and number of events, a multivariable analysis regarding prognostic factors was not possible since it was deemed to potentially lead to unreliable results. We were thus limited to univariable analysis only [24]. Nevertheless, patients requiring systemic therapy, thus lacking systemic disease control, or having two or more metastases at the time of SBRT treatment had worse PFS. This is consistent with the conclusions of Farooqi et al. who found that the most important predictor for improved outcomes is systemic disease control [4].

Despite these limitations and the relatively low number of evaluated lesions, a robust and significant relationship was found between dose and local control.

## Conclusions

In this study we show a strongly significant dose–response relationship between CTV median dose and local control with excellent LC rates for patients with metastatic lesions of sarcoma. The inhomogeneous dose distribution which generates higher doses than previous studies have reported could be an important factor to explain these results. The side effects using modern treatment technique were mild. We conclude that higher dose SBRT is an excellent and safe treatment technique to obtain LC of metastatic lesions of sarcoma.

## Data Availability

The data that support the findings of this study are not openly available and are available from the corresponding author upon reasonable request. Data are located in controlled access data storage at Karolinska University Hospital.
